# Selections and Abstracts

**Published:** 1902-09

**Authors:** 


### SELECTIONS AND ABSTRACTS.

### HOSPITALS OF THE CONFEDERACY.*

*Mr. President and Comrades of the Association of Medical Officers of the Army and Navy of the Confederacy:*

It is a great pleasure, independently of meeting each other, to find ourselves at the home of our dear old octogenarian comrade, the venerable S. H. Stout, M.D., Surgeon and Medical Director of the Hospitals of the Confederate Army and Department of Tennessee, who administered so successfully and brilliantly this great medical arm of the military service, so fortunately committed to him. Confusion very largely prevailed at first. The ablest surgeons and assistant surgeons, the most experienced, were in the field, while the hospital service was chiefly under the management and direction of the little experienced contract physicians, not immediately connected with the service. The required examinations that shortly followed as the service became better organized, weeded many of these out, and placed in charge of established medical posts, and in immediate charge of the Confederate hospitals, our very ablest surgeons and assistant surgeons.

Out of the chaos above described, soon order and perfect organization ensued, and the splendid hospital system which continued from this date uninterruptedly to the termination of the war, and for which result the chief credit is justly due to the superb administrative ability and tireless energy of our distinguished comrade and always friend, Medical Director of the Hospitals, S. H. Stout, M.D. Vastly more could and should be said respecting the official work of our beloved director of this great department, but time forbids, and I must pass on to the consideration of other points.

On the moment of an expected battle a telegram would be sent by the Medical Director of Hospitals to the hospital post surgeons within easy and rapid communication with the expected battle-field,

*Paper submitted by C. H. Tebault, M.D., Surgeon-General, U. C. V., of New Orleans, La., at Dallas Reunion for publication in the official organ.

to forward to the more distant hospital posts all the sick and wounded who could bear transportation, and immediately to telegraph for available supplies for the impending emergency. The able medical director in the field was always in instant official communication with the Medical Director of Hospitals. Thus there obtained no loss of time or confusion in knowing where to send the sick and wounded on such instant and momentous occasions, and hospital posts were thus always in readiness to receive and care for our wounded and desperately sick comrades whenever a battle was joined between the contending armies; and our unequalled women, God bless them all, likewise duly notified, were also prepared to provide with their own dainty and loving hands the needed delicacies possible that our straitened circumstances permitted.

When the wounded and sick were received, all who could stand a bath were given one, and those who could not were carefully sponged off, and all were dressed in clean cotton material for the bed and placed upon neat and comportable bunks. The surgeons and assistant surgeons, the nurses and our superb womanhood, were all now actively and zealously employed under a systematic and well-organized authority.

Our corps of nurses were detailed from the hospital sick and wounded who were convalescent, and were at once most faithful and efficient in the discharge of their duties under the close, vigilant and devoted eyes of our surgeons and assistant surgeons, who were unwearying in the performance of their tremendous and highly taxing duties. The prescriptions, written in a book kept for that purpose, were put up by soldiers detailed for that express purpose, but always under the direction and supervision of the ever-watchful medical officers of the hospitals. In all this important service the writer cannot recall a single instance in which an accident of any moment occurred, so careful and exacting was the supervision.

Again, on the eve of expected battle the near-by hospital posts would send on special cars a delegation from their medical staff accompanied by nurses, all of our nurses being men, and all needed appliances within our means and a supply of such delicacies as our precious and always ready women could prepare with their own hands for their loved ones in peril of death.

Thus we stood prepared for our responsible duties on the edge of the battle-field, ready to use our best skill there and then in caring for the wounded as they were being conducted to the nearest hospital posts.

Every hospital had its officer of the day, who was in authority on that day. Each hospital surgeon and assistant surgeon discharged in turn this important office, and concluded his day's inspection with a written report which was strictly examined by the surgeon in charge of the hospital, and then transmitted by him to the surgeon of the hospital post.

The surgeon in charge of the hospital had highly responsible duties; he received and disbursed all money under proper vouchers, looked after and cared for all hospital belongings, drugs, instruments, etc., bought all provisions and made regular monthly reports to the surgeon of the post.

The surgeon of the post supervised the entire hospitals at the post, received and gave all post orders and transmitted the hospital reports to our vigilant Medical Director of Hospitals. The assistant surgeons were the real active workers among the hospital sick and wounded; they were in immediate charge of the most commanding obligations and responsible duties, and excepting the associated duty of officer of the day, were restricted to these two duties of unsurpassed gravity and importance, and which, together with being members of the examining boards, claimed their well-nigh ceaseless occupation. Chickens, eggs, butter, vegetables, etc., were foraged for by convalescent soldiers detailed for this purpose, who frequently were out for a week at a time. Towards the last it was necessary to trade off certain articles valued by the country people, which were supplied us at these posts, as our money became valueless.

In the Confederate Hospitals it was the daily custom when the weather permitted to remove all bedding from the building and expose it to the sun and air. Those too ill to leave their beds were carried out in their bunks and placed in the shade of the hospital, or in the sun if desired, or under the shade of trees. Thus the hospitals were frequently cleaned and scrubbed and thoroughly ventilated and kept neat and sweet.

Hospitals frequently conducted vegetable farms by renting land in near reach, which was worked by the convalescents; and fresh vegetables thus secured for the sick and wounded. Every day committees of Southern ladies would visit the hospitals, bringing the delicacies wrought by their ever busy hands, and as they passed from bunk to bunk, speaking kind, cheering and comforting words to the occupants, and so ministering as loving and Christian and especially only Southern womanhood could.

All this was done with the utmost harmony and under the advice and directing eye and in hearty concurrence with the medical officers in charge. Lady matrons were connected with every hospital, and discharged their assigned and invaluable duties with the utmost efficiency and devoted faithfulness. In many instances they were of the very best families of the South driven from their desolated homes, refugees in consequence.

The strict professional courtesy and co-operation between the members of the medical staff, whether in hospital or field service, was a matter of wondering animadversion. The medical men of that day were men of the highest honor and standing at home. Their social rank was among the first; their unvarying unity of purpose; their social intercourse; their official relationship; their strict attention to every duty; their strict obedience to recognized necessary authority; their unbroken friendship and respect one for the other whenever they met; the complete and perfect harmony with which they worked together in the discharge of every duty assigned them, was the marvel and always favorable comment of all other departments of our military service. Their great-hearted, broad-minded humanity needs no better epitaph than this:

With 50,000 more prisoners of war to care for, prisoners of war whom the Federal Government refused to exchange for Confederate prisoners held in Federal prisons, and knowing the very scant and limited resources of the Confederate Government to properly provide for them, still in the face of this never-to-be-forgotten fact, the Confederate medical staff lost 4,000 less of Federal prisoners than the Federals lost in prison of Confederate prisoners whose exchange they persistently refused.

Smallpox cases were treated in tents properly floored, with due regard to drainage, and for heating when necessary, strictly and inflexibly isolated under wise and well-guarded precautions. The surgeons and assistant surgeons assigned to such encampments were restricted to these patients.

In the treatment of erysipelas and hospital gangrene, I can recall but a single instance at any one of our hospital posts where a distinct and separate hospital was assigned to all such cases occurring in the hospitals of a post, for the exclusive care and treatment of these cases.

The rule was to treat all such cases just where they occurred, in the same ward where the other wounded were cared for, and this was done with singular success and without marked detriment to the healthy wounds.

One blessing we enjoyed due to the blockade was the absence of sponges, clean rags being substituted for them with telling advantage. The rags could be thoroughly washed, as was done, and used over and over again. It is next to impossible to easily, if possible at all, to wash an infected sponge so as to render its employment safe again. This fact and the unstinted use of a plentiful supply of pure well or spring water, and the pure condition of the air of the hospital as above explained, were not without their wholesome effect. The healthy wounds were washed very generally with an infusion of red oak bark, a most valuable treatment. They were often stimulated with a brushing over with the tincture of iodine and were always well-nigh hermetically sealed by water dressings on which fresh water constantly dripped except when being dressed. The way the cases of erysipelas and the gangrene cases were healed caused them to be almost hermetically sealed also.

I now pass to the single instance in which these two dreaded maladies were treated in a separate and distinct hospital consisting of an encampment of tents. The single instance in my experience was at the post of Griffin, Ga. Here, at my suggestion, all these cases were sent to me and treated in the tents just mentioned and located within easy reach of my other hospital duties. It is worthy to record here that none of my other numerous wounded patients contributed to this encampment, though I was its surgeon as well. All my infected cases came from the other hospitals of the post, though their surgeons had no contact with this encampment; or came infected from the field of battle because of the tappings of roads or other unavoidable detention in transit.

This opportunity gave me a large practical experience in the treatment of these so much-dreaded complications by surgeons in both civil and military life. I can only speak briefly and generally, but I shall hope intelligently, on an occasion like this. When erysipelas had proceeded to the formation of pus, free incisions were made for its ready escape and these openings were syringed with properly diluted chlorinated water solutions, properly weakened tincture of iodine solutions, or solutions of tannic acid, once, twice or thrice daily, according to the severity of the case. The local application found superior to all others was the camphorated oil (not the linimentum camphoræ), with more or less sugar of lead added occasionally to meet special indications; but usually only the camphorated oil alone freely applied. This proved a cooling, salutary and healing evaporating lotion, and could be applied without restriction to any part of the body, even over the eyes when carefully protected.

The internal treatment consisted in giving quinine in pill form in which glycerine was incorporated to facilitate its absorption. These were administered in doses of two grains every one, two, three, or four hours to meet the febrile manifestations, and also for its well-known antiseptic properties. The bowels were, of course, carefully looked after. All complications were given their appropriate treatment. Let me note here that the general treatment of the Confederate Hospital Corps was aseptic not antiseptic, on the principle that an ounce of prevention was worth more than a pound of cure. That to prevent the possible diseased movement was better than to permit its occurrence and then strive to overmaster it.

The diet was eggs, milk, concentrated broths, and stimulants to meet the requirements of each case. This treatment gave most satisfactory results; together with an abundance of pure outdoor, fresh air. Occasionally for a short time, where pain was a factor to be prescribed for, an opiate would be exhibited, say, preferably, gum opium in grain doses every one, two, three, or four hours, or at longer intervals, to control this symptom, and given no longer than absolutely required. The gum opium was better tolerated than morphine, and did not disturb the stomach or appetite as obtained under morphine.

This treatment, thoroughly enforced in my hands, left nothing to be desired by me, and the local treatment mentioned protected against possible infection to others not so attacked. The best treatment for hospital gangrene was found to be the same internal medication, nourishment and stimulants mentioned for erysipelas, with fresh outdoor air in abundance. These patients were taken out on their bunks and placed in the open air under the shade of trees where they remained during the daytime, weather permitting. The local treatment was as follows: In the gangrenous stump, for example, cotton saturated with turpentine short of dripping was pressed into the gangrenous mass, completely plugging it. Over this was bandaged a poultice thus made: pulverized charcoal prepared fresh at the hospital, carrots reduced to a pulp, with hot water and flaxseed meal when procurable, but generally cornmeal in suitable proportions. These dressings were changed three or four times during the day and night. It was magical to see how soon the rotten mass would melt away, leaving behind a healthy wound to be subjected to the treatment of such wounds. It is impossible for me to enlarge or be specific in such a paper as I am writing, The healthy wounds were generally treated with solutions of tannin, or tincture of iodine, or of red-oak bark infusion, with which they were liberally and unstintedly washed as often as each case demanded. The lips of the stump were brought together, after thoroughly brushing over their interior parts to be healed with raw turpentine to stimulate the healing process—I say after this treatment the lips were brought in contact with strips of adhesive plaster of that date, which would hold to the satisfaction of any one, when first lightly brushed over with the oil of turpentine. Besides keeping the wound well united, it also furthered the healing in a wonderful degree, and no wound thus treated took on gangrene or erysipelas. Over this adhesive dressing cloths were fastened and either saturated with camphorated oil or kept unfailingly wet by the never-ceasing water contributed from suspended bottles devised for this purpose; but whichsoever was adopted, it required no failure of the medicated oil or the unceasing supply of water. These stumps were redressed from time to time as required, and whenever this was needed they were syringed out through any inviting aperture with one or the other of the three solutions referred to above, and the same outward application maintained until a complete healing resulted. One more experience and I will conclude this contribution. The best treatment for compound fractures in my experience was to treat them as though they were simple fractures. First, the part was thoroughly cleansed with a free supply of soap and water, then with the oil of turpentine, which again was thoroughly removed by ample washing with soap and water, so that not a vestige of the turpentine remained. Now the fracture was bandaged and adjusted precisely as for simple fractures and then starched; the wounds were thus hermetically sealed up. In a large wound, it was brought together with the adhesive strips as above prepared prior to bandaging and adjusting, with an opening left by the strips for the exit from the wound for any accruing discharge. This done, the bandage was applied and starched and the wound otherwise sealed by bandaging. This bandage was not removed until the fracture had united or until the requisite time had elapsed to justify the removal of the splint and bandage. Whenever the bandage showed any soiled spot due to the discharge from the wound, without removing bandage or splint it was thoroughly washed with warm soap and water and then with a weak solution of chlorinated water and again restarched; and this was done as often as a stain appeared or the faintest improper odor obtained. This treatment of sealing up was most perfect and safe in its results. There is a reason, and a most valuable reason, for thus treating wounds, as will immediately appear.

Our greatest of all sweeteners and purifiers are fresh air, water and sunshine, but there is a constituent from which the open wound must be carefully guarded from for any length of time as a rule. Oxygen is the element to which I here allude. It is the active principle of the atmosphere and is destructive in all its effects. Comprising one-fifth of common air, it is all around us for a chance to spring upon and devour something. We gather a basket of luscious peaches and put them out of the way of the children, but we do not outreach the sliest pilferer of them all—the oxygen—and we soon find the fruit covered with the prints of invisible teeth. Black spots appear, and we say they are decaying; it is only the oxygen feasting upon them, and in a short time it will devour them, skin and all. To prevent this, we put our fruit in a glass or metal can, heat it to expel the oxygen, seal it up tightly, and then it is safe from this chemical plunderer. We open the damper of the stove, and the air rushing in, the oxygen immediately attacks the fuel. Each pair of atoms catches up an atom of carbon between them and flies off into the air as carbonic acid. An animal dies. The oxygen is on the alert, and the instant the victim expires, and some times a little sooner, this agent, so anxious to commence, begins by removing that which would soon be an offense to all sensitive nostrils. We accidentally cut our finger, and soon the unwelcome oxygen begins at the quivering nerve beneath. The keen throb with which an unexpected hollow in a tooth is revealed to us announces the entrance of this foe at an unguarded breach.

It was the practice of Confederate surgeons and assistant surgeons to hermetically seal up all wounds as soon as practicable, and it was the almost universal adoption of this surgical procedure in all wounded cases that yielded us our splendid results in wound surgery. One word more about that splendid remedy the oil of turpentine, and I will conclude my paper. Applied pure to a healthy stump just amputated it will not only check all bleeding, but soothe the raw surface and immensely stimulate its more certain and rapid healing. It is equally soothing to mucous surfaces, but its contact with the skin is always irritating, as is well known. The other valuable use just alluded to, however, is not so generally known. I should not close without mentioning that our hospitals consisted of such large and commodious buildings as were procurable, as, for instance, school-houses, colleges, hotels, court-houses, churches occasionally, stores, and other like buildings suitable for the purpose, and all of which were patriotically tendered for this use. Small and large tents were everywhere employed to meet the wants of house accommodations. Rude, single-story buildings, for hospital use, raised some three or more feet from the ground, were also erected at given points in considerable numbers, and were constructed under the thoughtful directions of the Medical Corps, and were marvels of comfort under our trying surroundings, noteworthy for their perfection of ventilation. Apart from the side windows, which were ample in number, but without sash or glass, as we could command none, small, yet sufficient openings were provided at the floor surface and at the high ceilings, and capable of being closed or opened at will. Near the sides of the wall to the right and left of these buildings, one-fourth of these bunks were placed headboard against the sides of these hospitals, and the other three-fourths with headboards towards each other in the central space. These buildings were wide enough to allow a broad passage-way to the right and left extending through the length of the buildings between the footboard of the walls and centrally located bunks. I will make record at this place, for fear of omitting to do so, that chloroform was our universally accepted anesthetic for all purposes when one was needed, and was found far superior to ether from every point of view, and in all my hospital experience I can recall no death from its employment.

All medical supplies and instruments, and everything else required by us, were made contraband of war in spite of the immense number of prisoners of war held by us, and whose exchange was persistently refused on the part of the United States government. These hospitals were furnished with from forty-eight to sixty-four bunks with well calculated distances between them. They contained but two doors, a large front and a large rear door, and at the rear end two or more small rooms for the necessary hospital use. The bedding and all other material for equipping these bunks and all other washing and bed material were kept by the hospital stewards in separate rooms disconnected from these wards, and the most rigid rules of cleanliness enforced. The laundry work was performed some distance from the hospitals, and the cooking also was done at a proper interval from the sick and the wounded, and these and all else of importance were daily closely inspected and reported upon as stated, by the medical officer of the day.

The subject, my comrades, upon which I have addressed you, and which I now conclude, is necessarily, because of its vastness, simply skimmed upon the surface. It teems in all its depths with untold interest and is a harvest-field of immeasurable wealth to the possible medical explorer. When Richmond fell into the hands of the enemy, the fire which ensued destroyed the medical buildings of the Confederacy and its contained medical records were consumed. Fortunately our comrade, Medical Director Stout, preserved duplicate copies of the records of his department, and these are yet available for the student of that period of our history. Scattered records of the Trans-Mississippi Department may also be found here and there among the papers of the medical staff who here served during the war. All of the medical records of the Department of Northern Virginia must have been lost during the Richmond fire. The few of us now surviving cannot hope to do more than offer very imperfect sketches, as I have attempted to do in this contribution, in honor of the memorable work grandly, ably, and patriotically performed by our dead and living comrades of the splendid medical corps of the great Confederate armies.—*Southern Practitioner.*

---

### AN EXALTED PROFESSION.

### PHYSICIANS OVERWHELMED WITH HONORS AND DIGNITIES BY MONARCHIES AND REPUBLICS.

[From the New York Tribune, June 6, 1902.]

The present era seems to be the golden age for physicians. Every country appears to be bent upon overwhelming them with honors and dignities of one kind and another. In the list of honors granted ten days ago by King Edward on the day set for his coronation, the medical profession figures more largely than any other calling. A physician has just been intrusted by the President of the French Republic with the duties of premier, while here in the United States we find a medical man on the high road to the command of the army, after having filled with such signal success offices of exceptional difficulty, delicacy and responsibility as to lead to his being publicly extolled by the Chief Magistrate of the

Commonwealth as the most valued and valuable of the latter's servants.

It is difficult under the circumstances to realize that as recently as the early part of the reign of Queen Victoria physicians had virtually no place in European society. They were merely tolerated when eminent, and neither in London, nor yet in any of the leading capitals of Europe, would members of the great world ever dream of inviting their doctors to their dinner parties. Their position was indeed most invidious. To put the matter in plain English, they were not regarded, socially speaking, as gentlemen. Nor did they receive treatment as such. This was due in part to a survival in the olden days when the surgeon and the apothecary stood on the same social level as the barber, the three offices being often united in one and the same person. It was due, also, to the very natural awkwardness which a hostess might feel in receiving at her table on terms of social equality the medico whom she had feed in the morning and consulted about her health. All these ideas, however, belong to a bygone generation. To-day we find doctors being ennobled by nearly every one of the sovereigns of Europe.

There are some two-score baronets and knights in the medical profession of Great Britain, as well as a peer in the person of Lord Lister. Emperor William has conferred patents of nobility upon Dr. Ernest Leyden, the famous German physician who attended the death-bed of the late Czar; upon Dr. Koch, of bacillus celebrity, and upon a number of other stars of the medical profession in Germany and abroad. To the Regent of Bavaria belongs the credit of having converted Dr. Röntgen, the inventor of the X-rays, into a full-fledged baron. In republican France physicians have in late years received the highest grade of the Order of the Legion of Honor, and not only several princes of royal blood have adopted medicine as a calling destined to enhance their usefulness to their fellow-citizens, but there is likewise a full-fledged Queen, namely, the French consort of King Carlos of Portugal, who, having passed all the requisite examinations, is now entitled to add the letters "M.D." to her name, in addition to the word "Regina."

That this should be the case need furnish no ground for surprise. On the contrary, it is astonishing that so many years, nay, even centuries, should have elapsed before the medical profession came to be appreciated at its true worth. It is a calling to which all others, save those perhaps of a religious character, must necessarily give way. For even the greatest and most powerful in the land are not exempt from human ills, and when prostrated and rendered helpless by sickness or by physical injury, we are compelled to yield obedience to the directions of the physician, no matter what his birth or origin. There is no empire more vast in extent than that of Great Britain. Yet during the last fortnight the ruler of more than 300,000,000 of human beings, embracing one-fifth of the population of the entire globe, has been obliged to submit in the most absolute fashion to the behests of his medical attendants, who have not hesitated to exercise their authority in the fullest measure, realizing the burden of responsibility that rested upon their shoulders. It has not been merely that their professional reputation was at stake—for any error of a disastrous character in the treatment of a patient such as King Edward would entail, of course, the ruin of their medical careers—but it has likewise been because they have felt that their countrymen, and not alone their countrymen, but the entire civilized world, have looked to them and to their skill to avert the domestic and international crisis that is necessarily involved by the death of the executive of a great power, be he emperor, king or president of a republic. So strongly do physicians realize this that in several instances where mistakes in the treatment of their royal patients resulted fatally, they took their own lives, and I need merely remind my readers that, not even the suicide of Sir Richard Croft was held by his fellow-citizens to have constituted an atonement for the sorrow and trouble in which he had plunged the entire nation by the fatal error of his treatment of Princess Charlotte of Wales, only child of King George IV., and at the time of her premature death the heir to his throne. And within the last decade we have witnessed the self-destruction of one of the late Czar's physicians, while a celebrated colleague of his, who was held by the Russian people to be equally accountable for their ruler's death, had his house at

Moscow completely wrecked by the mob, narrowly escaping with his life.

The task of physicians in attendance upon rulers and upon royalty is rendered additionally difficult by the fact that until their illustrious patients become seriously ill and more or less helpless, they are proverbially self-willed, opinionated and somewhat touchy on the score of their health. They do not relish being told that they are ailing, nor do they like to obey directions with regard to precautionary measures in the way of diet and care until they are actually stricken. Moreover, some of them are in the habit of placing all the blame upon the shoulders of their physicians, whenever by any chance through their own carelessness and their own neglect to observe certain rules and regulations they fall ill. Finally, there is that pronounced objection which they one and all seem to entertain to permitting the public to be made acquainted by the doctors with the real condition of their health. And it is more often the fault of the distinguished patient than of the physicians and surgeons in attendance that optimistic bulletins are issued long after all hope has been abandoned, and up to the very moment when grim death is actually knocking at the door of the sick chamber.

It is only by means of the exercise of a vast amount of tact, discretion, courage and firmness that these doctors of personages of the rank of King Edward can manage to fulfill their duties toward their charges, as well as toward the people interested in the welfare of the latter. And the combination of these qualities is sufficiently rare to render those who display them valued, not merely as medical attendants, but as warm friends and trusted advisers. There was no one, for instance, who exercised so great an influence over the late Czarina of Russia as old Dr. Botkine, who, when first summoned to the imperial palace, had shown sufficient independence to refuse to diagnose her case—it was consumption—until she disrobed sufficiently to admit of the medical examination of her chest and lungs. He was quite young, utterly unknown at the time, and, by his sturdy honesty and decision of character, not only succeeded in getting her prudish majesty to do what all his more eminent confrères had previously sought in vain, but likewise inspired her with such confidence and regard that she retained him for the remainder of her life as her physician and best friend.

Only those acquainted with the inner history of the English court during the latter half of the Victorian reign are aware of the immense influence which Sir William Jenner exercised over the late Queen. It was an influence acquired as a doctor, but which led to his advice being sought by his illustrious patient about all sorts of matters, many of them entirely foreign to her health. He was wont to see her regularly twice a week wherever she happened to be, these visits never, however, being recorded in "The Court Circular," which she herself edited; and, Sir James Reid, the resident physician to her majesty, being her regular medical attendant, the visits paid by Sir William Jenner to Victoria must be regarded as having been rather those of a friend and adviser than as professional calls. Sir Francis Laking is on the high road to attain a similar position with regard to King Edward, who not only values his science, but shows a marked predilection for his companionship.

The baronetcies conferred upon him, as well as upon Sir Frederick Treves, who performed the recent operation upon the King, are likely to be supplemented, in the event of the latter's complete recovery, by further honors, which will probably take the form of peerages, thus adding to the number of medical lords in the upper house of the national legislature. Nor will any fault be found with such promotion. The days when the English press and people united in abusing King George IV. for knighting his doctor, and for confiding to him the duties of private secretary, as the only man upon whom he could absolutely rely, are long since past. Gone, too, are the days when *Punch*, with ponderous humor, ridiculed the idea of granting hereditary honors to medical men by putting forward a suggestion to the effect that the physician in attendance at those events in Queen Victoria's family which are described as "happy" in the palace as well as in the cottage, should be raised to the peerage under the title of "Lord Deliverus," and not a word of objection will be heard to the addition of two such valuable recruits to the upper house of the British legislature.

The extent, indeed, to which the medical profession has raised itself in the eyes of the people may be seen by the fact that when, the other day, the *London Lancet* recommended that one of the three boys of the Prince of Wales should be reared as a physician, giving as its reason that the English medical profession "has long merited acknowledgment, and the finest acknowledgment would be the enrollment in their body of a prince of the blood," the idea met with wide-spread expression of approval, several notable cases on the Continent being recalled where royal personages have taken the trouble to acquire this, the grandest of all callings, and to render themselves more than ordinarily useful to their fellow creatures, the late Empress of Austria's brother, for instance, being able to claim to have restored the sight to more than three thousand people from whose eyes he has removed cataracts during the last twenty years.

The increase of prestige of the medical profession in late years may be said to have likewise extended—though of course in a minor degree—to the calling of sick nurse, and the late Empress Frederick, the late Queen Victoria and Queen Marie Amelie of Portugal, but more particularly Queen Alexandra of England, have all four contributed in no small measure to improve their position from a material, a scientific and social point of view. But while the era of "Sairey Gamp" is a thing of the far past, yet much still remains to be done, especially in keeping the ranks of the trained nurses free from those oft well-educated adventuresses who assume, along with the garb, the frequently borrowed credentials of a trained nurse in order to worm their way into families, upon which they subsequently prey, but into which they could never have found admission save in the guise of that profession ennobled by Florence Nightingale, who still survives, honored and respected throughout the civilized world. Sick people are so completely at the mercy of the nurses engaged to tend them, and there have been so many cases revealed in the law courts where the more or less pseudo-nurse has taken advantage of the helplessness of her charge, that the utmost care should be taken alike by the doctors and relatives of a sick person to assure themselves of the character and antecedents of the nurse before confiding the patient to her hands.

EX-ATTACHÉ.

### THE RELATION OF THE EXAMINER TO THE AGENT.*

If one look casually at the machinery of the life insurance business, he may think that the doctor is intended to be a check upon the agent. I refuse to accept any such superficial or unjust judgment. It is unjust because it implies that, as a rule, the agent needs to have a check or a safeguard on his business. A long and wide experience has convinced me that the average morality among all civilized men is much the same—that agents are generally as good as doctors—and that doctors are generally as good as ministers. I can attest that in my fifteen years' experience as a medical examiner, never once did an agent attempt in any way to corrupt me or in any degree to make me disloyal to my company. And in the comparatively few instances of insurance fraud that I know of, if there was a corrupt agent in the deal, you may feel sure that there was also a corrupt doctor at his side. The truth is, gentlemen, that we all, examiners and agents, are simply fellow-workers in a good cause. And it is a good cause, a splendid vocation—for life insurance is one of the beneficent developments of civilization—it is a practical carrying out of the apostolic injunction, "Bear ye one another's burdens."

Being fellow-workers, it behooves us to help each other in our work, and to recognize and bear with each other's necessary limitations. This is what I understand by the subject assigned to me, viz., "The Relation of the Examiner to the Agent."

In their published instructions, not a few of the companies are explicit in their statements as to the relation of the examiner to the agents.

The Equitable Life says: "Agents have no concern whatever in the premises—neither in the procuring of appointments nor even in the making of original nominations for vacant examinerships, except by courtesy. Examiners will therefore understand that they hold office from the medical and not from the agency department of the society—and that they are consequently responsible to the medical department only. Agents are not authorized to direct examiners concerning their duties."

*Read by Dr. David J. Doherty, of Chicago, Ill., before the Chicago Life Underwriters' Association.

The New York Life says: "The examiners are in no sense dependent for their appointments, dismissal or fees upon either applicants or agents. It is to the interest of all that the relations of the examiner toward both the applicants and agents should be cordial and friendly. . . . While it is often the duty of the examiner to oppose his judgment to the wishes of agents by refusing to recommend some of the applicants for insurance, yet he ought, by the exercise of tact and judgment, and especially by firmness in his convictions, to avoid all friction and thereby secure that harmony so essential to a pleasant and profitable intercourse and to satisfactory business results."

The Northwestern Life says: "The examiner will under no circumstances permit personal friendship for the applicant or the agent to influence him in giving the company a fair and candid opinion as to the desirability or undesirability of each subject he examines."

The Union Mutual says: "Medical examiners are appointed by the home office, and are consequently independent of the agent; but it is for the interest of all concerned that harmony and concert of action should exist between the agent who solicits and the physician who examines the risks."

From these quotations it is evident that all companies desire that there shall be no friction or interference between us—that there shall be, on the contrary, cordiality, friendliness and harmony; that both examiner and agent shall do good work—each in his own sphere.

These results will follow, gentlemen, if we respect each other—and we will respect each other if we know each other—if we recognize the qualities that each have, the duties each must perform, and the limitations necessarily inherent in human nature.

An examiner must possess professional skill and experience, integrity of character, soundness and independence of judgment, and industrious reliability. These are all manly qualities, and are entitled to the respect of all fair men. He is the trusted and confidential adviser of the company and the guardian of its interests—a position that is certainly entitled to the respect of all fair men, and especially of all loyal fellow-workers for the company.

The qualifications of an agent should be at least those of a good business man—well posted in his line of business—and these qualities are knowledge, integrity, industry and enterprise; qualities that deserve and receive respect everywhere. To my mind, the ideal agent ought to be more than an A-1 business man. He ought to see the humanitarian and philanthropic side of his work, and should be impelled, not merely by the incentive of commissions, but also by zeal for the welfare of his brother-men. The splendid motto of this Underwriters' Society, "*Non Solis Nobis*," means just that, if I understand Latin. Not for yourselves alone do you work, but for your fellow-men, and better still, for their helpless wives and children. Since, then, gentlemen, we must be friendly fellow-workers, let us have sympathy for each other. The work of neither is too easy. Your work is hard in spite of the self-evident proposition that all men ought to be insured. It is self-evident that all men ought to be good, but ministers have no light task to make them so. It is self-evident that all men ought to be healthy, but doctors find it very hard to induce them so to live as to remain healthy. And so, likewise, it is self-evident that all men ought to be insured, but it often requires much planning ahead, maneuvering and diplomacy to induce a man to take insurance. The transaction often involves rebuffs and disappointments, and then, if made, it must be steered between the Scylla of the medical examiner and the Charybdis of the home office. There is much anxiety and many a slip 'twixt cup and lip before the little bark is safe in port, the policy delivered and your commission tucked away safely in your inside pocket.

It is indeed exasperating for an agent to see his case turned down, and we give you our sympathy, but on the other hand our work is not easy, and we also ask your sympathy.

You are aware that theoretically examinations are made leisurely and at convenient hours in a private office, either the doctor's or the company's. Practically, as a result of the intense competition for business, most examinations are made at the applicants' homes, at inconvenient out-of-business hours, and amid unfavorable surroundings. The doctor needs your sympathy. He is often tired by the rush of work, exasperated by vague or incorrect addresses, provoked by refusals or by failures of applicants to keep appointments; and he must ever carry with him the weighty responsibility of the company's welfare. If his own honor would let him escape it, a lynx-eyed home office will hold him closely to it.

I do most of my examining on Sunday forenoon, because it is the best time. As I drive about, I see the people trooping out of church with their glad Sunday faces and bright Sunday clothes, and I pity myself as an Ishmaelite cast out, or a Moses who can see the golden land of Sabbath Peace only from afar. Sometimes I meet very hard luck, perhaps a whole forenoon spent, and my stock of cases only diminished by two or three. You have heard of the man who went up on a mountain to pray for rain and got struck by lightning. That was hard luck. Well I sometimes almost envy him—so tired and disappointed am I—and an exasperated agent might well pity and forgive me.

The doctor should give to the agent a reasonably prompt service and accurate work. The doctor should do more than that. It may often occur that a timely word from him will decide a wavering applicant. He ought always to say that word, and I believe that doctors frequently do that—we are glad to help the company to get a client and you to make a commission, and we don't want any rake-off.

But, gentlemen, there are some things that the agent ought to do, and other things he ought not to do or expect.

First. He should make reasonable appointments. He ought to know the doctor's office hours, so that the applicant can call upon the doctor during his office hours, or the doctor can call upon the applicant outside of them. In important cases, of course, the applicant's convenience takes precedence of everything. We all understand that in the case of any important man, we would drop everything to go to him. But, as a rule, among business people, who nowadays are almost all provided with telephones, it is usually best to let the doctor call up the applicant and let them both make their own engagements. For other people, employees who cannot control their own time, all the doctor needs to know is when they are usually at home, and he will catch them. I find that that class of people usually forget or do not keep appointments which are made for them or by them.

Second. The agent ought to give accurate directions. That does not mean street directions, in which there is seldom an error, but you know that in this great city of flat-dwellers, the doctor needs to know the number of the flat, the floor, and whether front or rear, and such definite directions that he can go direct to the applicant's door. For ten years I have preached, prayed and begged for this, and yet the agents will not put it down. I was glad to see the other day that Mr. Cleveland said it was proper for a fisherman to say "Damn," because, while I can say, with Mr. Cleveland, that I was raised a Christian, I never did use cuss words until I got into the insurance business, and then the only time I use them is upon occasions like this: when, about 9 p.m., I arrive in front of a large flat building in which I wish to find John Smith, and no floor nor flat number is given, I fumble for my glasses and get them on, and I light a match and look along the letter boxes, and the chances are that there are no names on them, and then I stumble up a flight of stairs, perhaps dimly lighted, if at all, and knock here and there, asking for Smith. It is not fair to the people, and it is not fair to the doctor, and the agents can so easily rectify that trouble if they will only take the pains to do so. Now, don't you think I have a right on such occasions to say "Damn the agent?"

Third. The agent should not ask the cause of the rejection of an applicant. It happens sometimes that the agent will come up and say, "Doc, what's the matter with that case I wrote down there? Do you remember?" Now, we will not tell. If we say anything, I can assure you in advance that it will not be the truth. It is a professional secret which belongs to the applicant, and if you were the applicant, you would not want the doctor to tell, and therefore you must not ask.

Fourth. The agent should not expect to be present at the examination, or attempt to advise or interfere with the examiner's work. I don't know of any instances of that kind, but I have read of them in the books. The agent should not expect to be present at the examination. I never met one who desired to be, but sometimes they do desire to look over the papers after the doctor has done his work. I think that is forbidden by all companies. Occasionally, for some reason or other, a paper has to be sent back to them, but I am always careful not to finish it, so that it must come back to me, and they can never learn from the paper what the medical judgment is.

Fifth. Agents should not offer nor accept a division of fees. I have never heard of any of them attempting that, and I will say now that the doctors do not divide fees. I don't know about the agents, but we don't; we hold on to all we get.

Sixth. The agent should not attempt to influence the examiner's judgment; but I have never known of that being attempted.

Seventh. The agent should not try to insure the doctor by offering him an examinership. That sometimes happens in little country towns, but whenever it does the doctor is a sucker, for he only gets one or two examinations and that's all.

Eighth. The agent should not expect the doctor to do the agent's work, just as little as he should do the doctor's work. Of course, doctors are always willing to fill an omission, such as a date, or something of that kind. When the agent says he forgot to get the exact date of the applicant's birth, we get it. But now wouldn't it "jar" you if you were a doctor and you got a paper like I had a few weeks ago? The application side was entirely blank, and it had a note pinned on it, saying, "Dear doctor, please fill out and get all necessary details and signature upon my side of this paper and bring it back to me, and we will fix it together before it goes." Well, it took me three trips to locate the applicant, and when I did, he said, "Why I never authorized anybody to take out insurance for me." That agent is perfectly honest, but I have not yet had a chance to have a row with him, and I have made no fuss about it. It is inexplicable to me why a sane man should do such a thing, or why he should want the doctor to do it. But you see it sometimes happens.

Ninth. The agent should not present bad risks. Of course, the agent is not required to make a diagnosis. He is not supposed to be a doctor. I have heard of but one case like that. That agent has a high position now in an insurance company in another city in the East. I remember well the occurrence. The case was so bad that the ordinary layman would say, "I am rather doubtful; I had better not waste any time on that." When the company wrote to him, his answer was, "I am not the doctor; I only present the cases, and you have the doctors to pass on them." Technically he was correct. Nothing was done, and nothing further was said; his position was logical. But, after all, it was not the best thing or the right thing to do, because the agent is also supposed to look out for the interests of the company, and not for his own alone.

Tenth. If the case is rejected, the agent should not blame the doctor. The tendency is always to blame the doctor. You have heard of the man who was arrested for robbing a till, and the judge said to him, "Why did you rob that man's till?" and he said, "Why, my doctor told me I must have some change." So we always blame the doctor. Now I will give you some figures. Since January 1, 1902, I have examined 324 cases, and the sum total would run up to near a million. Of those I rejected 24, or 7 per cent., and I rated 13 as "fair." The home office altogether rejected 13, postponed 6, and offered special class policies instead of standards in 9 cases. Now, that is not a very high percentage of rejections, if you will for a moment think of all the albuminurias, heart troubles and all the other troubles that flesh is heir to. The doctors go into the medical side of the applications, but the home office considers also the moral hazards, and both of these causes can easily produce the 15 per cent. rejections which seem to be about the average in all companies. The agent may present a thousand applications, but he does not in any way affect the company or jeopardize its assets. But as soon as the field examiner and the home director accept the case it is issued, and the company is at once obligated to that extent. That is the difference between your work and ours, and the reason why we must be much more conservative than you. I presume a large number of us belong to lodges. Now, in a lodge ceremonial you know that there is an outer guard and an inner guard. If I might apply that comparison to the life insurance business, I would say that the agent is the outer guard and the doctor the inner guard; they are not hostile to each other, but they are both friends, brothers, fellow-workmen; neither is trying to do up the company, but they are both watching its interests and building up its business, thereby benefiting both themselves and the world.—*Medical Examiner.*

---

### OBSERVATIONS RELATING TO SURGICAL TECHNICS.*

In appendicitis with well-defined retro-cecal abscess the lumbar incision advised by Edebohls should come into consideration. Edebohls's method will hardly be accepted for general application in appendicitis, but where the pus accumulation is distinctly retro-peritoneal the lumbar incision must commend itself as a very safe and convenient avenue of attack.

It is difficult to understand why one should not use a readily absorbable ligature material in closing hernia wounds. If the suture material be completely sterilizable, the fact that it is soft and promptly absorbable cannot be held to be an objection to its use in hernia. The suture does its work during the first few days, after which its presence in the tissues can only be productive of harm.

In using perineal drainage a greater fall for the urine from the bladder may be secured by elevating the hips upon a rubber air cushion ring. In addition to raising the hips, the ring greatly lessens the likelihood of the occurrence of bed-sores.

The writer knows of no method of retaining the large double-eyed perineal drainage catheter so effective as that of stitching the catheter with a silk ligature snugly to the upper angle of the skin wound. The catheter can neither slip backward and project too far into the bladder, nor forward, withdrawing the eye from the cavity of the viscus. When after a few days the catheter must be changed, an easy but less accurate means of retention may be used which consists in passing a safety-pin through the catheter wall, tying narrow tape bands about the catheter in front of the pin, using two ends for perineal straps and passing two ends upward over the pubis, all to fasten to a band of tape encircling the waist.

In closing wounds it is a mistake to use the continuous suture

---

*By Joseph Rilus Eastman, M.D., Indianapolis, Ind.

except in certain rather rare cases, where rapidity of execution of the operation is of vital importance. When there is tension upon the wound edges the continuous gut suture is objectionable, for the reason that softening and dissolution of the gut at any point along the wound line during the first few days means the tearing apart of the whole wound. Likewise infection of the gut or silk at any point means infection all along the wound line. If for cosmetic reasons it is desired to use a subcuticular stitch, it should not be forgotten that interrupted sutures may be thus buried as well as the continuous and with less danger of sudden separation of the edges of the wound.

Whereas local anesthesia suffices in nearly all cases of circumcision in adults, it almost invariably disappoints if employed for this operation in children. Neither Schleich's infiltration anesthesia nor cocain removes the dread of pain and the shock occasioned by the sight of blood. There is moreover good reason to doubt whether purely anesthetic effect of cocain is as pronounced in babies as in adults. Indeed, instances of the production of actual local hyperesthesia by the subcutaneous injection of cocain in children are not rare. A general anesthetic is therefore to be preferred in the performance of circumcision in children and in the writer's belief the best and safest for use in children is the sulphuric ether.

The most satisfactory dressing after circumcision of babies and young boys is prepared by thoroughly annointing the parts with carbolized vaseline and applying immediately over this a three-cornered piece of oiled silk, the base attached above to a narrow tape encircling the waist just above the hips and the apex attached to two perineal straps of tape which fasten behind to the belt tape. The author has found this dressing quite safe and grateful to the very young patients, since there is nothing used which may stick to the wound, causing pain and destruction of granulations upon removal.

In irrigating the urinary bladder, as in acute or chronic cystitis, the catheter should be discarded wherever possible. It is only rarely that the irrigating fluid will not pass back under comparatively low pressure from a blunt nozzle at the external meatus into the bladder, the patient meanwhile relaxing the compressor urethra muscle by making effort at micturition.

Von Brunns and Powell believe that they have found in pure carbolic acid a disinfectant for infected wounds which will disinfect without injuring the tissues. In infected wounds after occasionally mopping with small gauze pads dipped in pure carbolic acid and immediately washing in alcohol, it was observed that the wound secretion was much reduced, and that instead of dressing the wound twice a day a change of the dressing every third or fourth day sufficed.

Powell regards alcohol as a perfect antidote for carbolic acid. That the alcohol neutralizes the acid so far as the escharotic effect of the latter is concerned, can hardly be doubted. The whitening of the tissues attendant upon coagulation of the albumen of the superficial cells is almost immediately removed upon application of the alcohol. In just what manner the alcohol restores the cell, the albumen of which has been coagulated, does not appear from the writings of Powell. The writer coagulated agg albumen with pure carbolic acid and found that the addition of absolute alcohol only hardened the coagulum. It is, however, well-known that in wounds the alcohol almost immediately restores the whitened tissues to their normal color. Perhaps the condition produced by the carbolic acid is not that of true coagulation necrosis as has been supposed. At any rate the carbolic acid seems to destroy or materially inhibit the growth of bacteria in the superficial cells of an infected wound, and the alcohol acts as an antidote to the destructive action of carbolic acid upon the tissues themselves. It may have occurred to some that the alcohol acts not simply as an antidote to the acid, but is hardly inferior to the carbolic acid as a destroyer of germs. It has been the practice of the writer to apply gauze pledgets with fifty per cent. alcohol over stitch abscesses and small superficial wounds generally. Such pledgets may be applied fresh several times daily for one or two days, and followed by the application of iodoform or any antiseptic siccative powder. The writer has found this a useful method of dealing with small and superficial suppurating foci.—*Medical and Surgical Monitor.*

### A DISCUSSION OF THE METHODS OF OPERATION ON THE PROSTATE.

In the Section on Surgery and Anatomy, at the recent meeting of the American Medical Association, Dr. E. W. Andrews presented a paper on the operative procedure on the prostate, and compared the advantages of the suprapubic and perineal routes. The disadvantage of each was that the urinary tract was opened. From this there was a considerable source of danger. There was great need of an extravesical method which should avoid this and make the operation a safer one. The prostate was held very firmly in position in the pelvis, and it seemed quite probable that the reason why some large prostates gave no symptoms, and some small ones caused marked difficulty, was that the bony walls of the pelvis prevented any motion backward and downward of the gland. After treating a number of cases in the ordinary way, he determined to try to invade the region through an anterior incision which should admit him to the pelvis via the pelvic angle. This he found to be quite practicable, and he had done three such operations, all of which were successful. The basis of his technics was entirely to free the prostate from its ligamentous attachments anteriorly and above, and to remove all the portion of the gland anterior to the urethra.

Dr. Ochsner, of Chicago, said that Dr. Andrews's paper had been of great value. He urged the use of the perineal incision, however, for prostatectomy, inasmuch as it gave admirable results if great attention was paid to technical detail in controlling hemorrhage.

Dr. Moore, of Minneapolis, said that Dr. Andrews had demonstrated a very important truth when he showed that obstruction was not in proportion to the size of the gland. He was not yet prepared to commend the proposed anterior partial prostatectomy. He desired to emphasize the importance of the preservation of the openings of the seminal ducts, because, as the operation became perfected, younger men would be more anxious to have it done. Dr. Andrews's route did not seem practicable to him. He would not condemn it, however, without trial, because it seemed theoretically the correct course to pursue, and might be capable of broad application.

Dr. Murphy, of Chicago, said that in all cases of enlarged prostate, the urethra, as it passed through the organ, was also markedly enlarged, often to the extent of reaching an inch in diameter. In the occasional cases of persistent urinary fistulæ, which followed the perineal operation, he had taken pains to find the cause. It was due to the downward dragging of the hypertrophied mucous membrane of the part which was left behind; this became loosened after the removal of the organ, and dropped down into the wound. He would continue to do perineal section in removing prostates, and agreed with Dr. Ochsner's mode of treating the hemorrhage. The so-called middle lobe was not a part of the prostate at all.

Dr. Ferguson, of Chicago, had given up the suprapubic route entirely. He believed Dr. Murphy to be entirely wrong, both in his conclusions as to the etiology of fistula formation and as to the size of the prostatic urethra. He reported 21 cases without a death, the patients' ages varying from 49 to 82 years.

Dr. Young, of Baltimore, expressed great astonishment that in this profoundly interesting discussion on the prostate, no one had as yet spoken of Bottini's operation. He had recently done the operation 55 times with 3 deaths, 2 of which occurred in patients who were moribund at the time of operation; 19 of these men were over 70 years of age. He admitted that the operation was not surgical, but stated that it had a broad field of usefulness, because, as yet, prostatectomy was done only in very grave and advanced cases.

Dr. Parker Syms, of New York, said that he was glad to see the trend of opinion well turned in the direction of perineal prostatectomy. He was one of the first to advise it, and had been patiently striving to introduce it for many years. He hoped in a few days to do this operation on a patient upon whom the Bottini operation had been done by a very competent surgeon no less than four times. He referred to his rubber bulb catheter, which, when introduced into the bladder and filled with sterile water, gave an admirable opportunity to make traction on that organ and thus pull down the prostate. He had operated on 20 patients by this method, all of whom had recovered without discomfort.

### CRYPTORCHIDISM, WITH A REPORT OF TWO CASES OF NATURAL EUNUCHS.*

H. G. Anthony, Chicago, reports two cases: Case I. Is an individual aged forty. He is mentally weak and has recently become extravagant. He never had an erection of the penis. His voice is puerile and has never changed. The breasts are of the male type; there is some hair in the axillæ; there is no male distribution of hair on the breast or abdomen; in the pubic region there are not to exceed a dozen illy-developed hairs.

The penis is the size of that of a boy of six years of age, but perfectly formed; the foreskin may be retracted; the glans penis is normally formed; the urethra is small, but normal and proportionate to the size of the penis.

The scrotum is also small, but perfectly formed, with a normal central raphe. No testicle and no epididymis can be felt in the scrotum on either tide. Passing the finger along the inguinal tract by invaginating the scrotum, no spermatic cord can be felt; the internal ring is not open; no testicle can be felt at the internal ring, and there is no impulse on coughing on either side. No ectopic testicle can be discovered in the usual location of ectopic testicles.

Case II. This patient, age twenty-six, is about five feet six inches in height; rather corpulent, with prominent abdomen. He attended school for two years when a boy, but he was not sufficiently intelligent to keep up in his classes. Beyond the fact that he never was intellectually bright, he showed no peculiarities until after puberty. He can write his name and simple sentences, and do easy sums in arithmetic. He never reads or studies. He is an inveterate smoker, and his mother finds it necessary to limit the number of cigars he smokes each day.

He has no occupation and never was sufficiently intellectual to engage in business. He spends most of his time in a cigar store, conversing with the proprietor and watching customers come and go. He has many habits which are disgusting to those about him. His table manners are such as to necessitate his eating separate

* Jour. A. M. A., August 2, 1902.

from other members of the family. His condition is generally known to his associates, who consider him a hermaphrodite, and assign to him many peculiarities of mind and body which he does not possess.

He states that he occasionally has a hemorrhage from the penis, losing about a teaspoonful of blood each time; the bleeding continues about one-half hour, and occurs at irregular intervals. His physician has observed one of these hemorrhages. His friends interpret them to be menstruation. The cause of the hemorrhages cannot be ascertained; it is to be suspected that they are due to some foreign body being purposely introduced into the urethra. The meatus is swollen, and the mucous membrane everted. The urine is voided in a good stream; it is normal and contains no shreds. There is no urethral discharge, and no induration can be felt along the course of the urethra.

The thyroid gland is enlarged to a moderate degree, the left lobe being larger than the right and very hard. This enlargement has been present as long as he can remember. His voice is eunuch-like, and when he speaks in a loud tone it is squeaky, like that of a youth during the time the voice is changing. He has no beard and never shaves. There is some hair in the axillæ; none on the breast or abdomen and no more than six or eight hairs in the pubic region.

He presents the female habitus of hand, arm, hips and lower extremities. The penis is not larger than that of a boy of six years, but perfect in its formation. The scrotum is perfectly formed, but also of small size. There are no scars on the scrotum which could be the line of incision of an early castration. The scrotum contains no testicles, but in the upper part of the right side can be felt a cord which is possibly a rudimentary spermatic cord. There is no ectopic testicle.—*St. Louis Med. Review.*

---

### HOW TO CONDUCT A NORMAL LABOR.*

The patient is examined frequently, the urine in particular being tested often for albumin during the latter part of the gestation period. Instruction is given in regard to diet, exercise, hygiene

* By James Moran, M.D., New York *Medical Journal*, May 31, 1902.

and clothing. Careful inquiry also is made into the history of her case. It is insisted that the patient be confined in a large and well-ventilated room, and a sunny one if possible. As to the preparation of the patient for labor, the writer believes in the same care as though she was to undergo an operation. The list of things needed by the patient and child include two pieces of oilcloth or rubber sheeting, six abdominal binders, four bed-pads, one pound of absorbent cotton, twenty-four vulvar pads, twenty-four baby napkins, one small blanket, a fountain syringe and a douche pan, two or three wash basins, one dozen clean towels, six clean sheets, olive oil, cake of pure soap, one nail brush, plenty of safety pins, three clean aprons, one pot of boiling water, and another that has been boiled and set aside to cool.

Before examining the patient the hands are scrubbed with as much care as possible. An examination under the bed-clothes is never made. Sterilized rubber gloves are used if recently in attendance upon any infectious cases.

Among additional things in an obstetric bag, the writer carries a double tenaculum, a uterine-dressing forceps, a square yard of iodoform gauze, a needle-holder, perineum and cervix needles, and sterilized sutures.

Small doses of morphine (⅛ grain) are given every hour or so where the woman suffers severely and labor progresses slowly. Chloroform during pain is given upon the head reaching the perineum. The limbs are kept extended during expulsion, the anesthetic pushed to the surgical degree, but discontinued upon the birth of the head. Pressure is made over the uterus for half an hour after the completion of the placental stage, all lacerations repaired at once, all blood-clots removed, a binder applied to the abdomen and a sterile pad to the vulva. Before and after every nursing both the mouth of the infant and the nipples of the mother should be cleansed with a solution of boric acid. After the second day it is advised that the patient lie for a short time several times daily upon the stomach, in order that any tendency to retroversion may be obviated, and to favor drainage from the vagina. The patient should remain in bed until the flow ceases to be red. A post-partum examination is made on the tenth or twelfth day.—*St. Paul Med. Journal.*

### THE EXCLUSION OF CONSUMPTIVES FROM FAVORED LOCALITIES.

The modern facility for segregating tubercular patients in definite localities possessing climatic advantages is one which has not been without its disadvantages. This has recently been recognized by a proposition on the part of some Colorado legislators to pass a law prohibiting the admission to the State of aliens suffering from tuberculosis. Still more recently it is reported that the hotel proprietors of the French Riviera have met together, and discussed discouraging the admission of tubercular subjects to their hotels. Of course nothing has come out of these propositions, as public opinion and private interest are as yet opposed to each other, and any harsh treatment of the tubercular subject would call down vials of wrath upon the head of the offender; but they serve nevertheless to show which way the wind is blowing, and they indicate that there is growing in the mind of the public, right or wrong, a conception of the danger attendant upon segregation.

The idea that tuberculosis is a transmissible disease is one which, as it takes root in the public mind, must naturally lead to many misconceptions. The difference between degrees of transmissibility is one not easily recognized, and it will be difficult to establish clear and definite distinctions of danger limits in the lay mind. The risk of infection, however comparatively small, will actuate many to avoid localities in which the subjects of the disease are accustomed to congregate. It is, however, this fear of infection which will ultimately bring about a clear understanding of the character of the *materies morbi*, the necessity of its destruction, and furthermore will slowly but surely tend to check the marriage of those of pronounced consumptive tendency. The fact that tuberculosis is everywhere endemic, and that its subjects can never be excluded from any locality, favorable or unfavorable for its treatment, should be apparent to those who with more enthusiasm than judgment seek to circumscribe themselves with protective barriers. It is morally certain that any action which would deprive these unfortunate invalids of common human rights would never receive the sanction or approbation of the public.

The best thing for those who are brought into actual sociological relation with consumptives, whether as landlords, legislators, or public officials, is so to order events that the public may be taught how to preserve an immunity against infection.

So too must the individual patient realize that climatic treatment is not a cure, but rather a means of instructing the invalid in good habits of life and of impressing upon him the necessity of introducing the hygienic life into the home.—*Medical Age.*

